# Distinct correlation network of clinical characteristics in suicide attempters having adolescent major depressive disorder with non-suicidal self-injury

**DOI:** 10.1038/s41398-024-02843-w

**Published:** 2024-03-05

**Authors:** Bo Peng, Ruoxi Wang, Wenlong Zuo, Haitao Liu, Chunshan Deng, Xiaoyuan Jing, Hongtao Hu, Weitan Zhao, Peiwu Qin, Lei Dai, Zuxin Chen, Yingli Zhang, Xin-an Liu

**Affiliations:** 1 Department of Depressive Disorders, Shenzhen Kangning Hospital, Shenzhen, Guangdong China; 2 Shenzhen Mental Health Center, Shenzhen, Guangdong China; 3 Guangdong Provincial Key Laboratory of Brain Connectome and Behavior, CAS Key Laboratory of Brain Connectome and Manipulation, Brain Cognition and Brain Disease Institute (BCBDI), Shenzhen Institute of Advanced Technology, Chinese Academy of Sciences, Shenzhen-Hong Kong Institute of Brain Science-Shenzhen Fundamental Research Institutions, Shenzhen, 518055 China; 4 CAS Key Laboratory of Quantitative Engineering Biology, Shenzhen Institute of Synthetic Biology, Shenzhen Institutes of Advanced Technology, Chinese Academy of Sciences, Shenzhen, 518055 China; 5 Shenzhen Longgang District Maternity & Child Healthcare Hospital, Shenzhen, Guangdong China; 6 Institute of Biopharmaceutical and Health Engineering, Tsinghua Shenzhen International Graduate School, Tsinghua University, Shenzhen, 518055 China; 7 Tsinghua-Berkeley Shenzhen Institute, Tsinghua Shenzhen International Graduate School, Tsinghua University, Shenzhen, 518055 China; 8 University of the Chinese Academy of Sciences, 100049 Beijing, China

**Keywords:** Depression, Human behaviour

## Abstract

Suicidal behavior and non-suicidal self-injury (NSSI) are common in adolescent patients with major depressive disorder (MDD). Thus, delineating the unique characteristics of suicide attempters having adolescent MDD with NSSI is important for suicide prediction in the clinical setting. Here, we performed psychological and biochemical assessments of 130 youths having MDD with NSSI. Participants were divided into two groups according to the presence/absence of suicide attempts (SAs). Our results demonstrated that the age of suicide attempters is lower than that of non-attempters in participants having adolescent MDD with NSSI; suicide attempters had higher Barratt Impulsiveness Scale (BIS-11) impulsivity scores and lower serum CRP and cortisol levels than those having MDD with NSSI alone, suggesting levels of cortisol and CRP were inversely correlated with SAs in patients with adolescent MDD with NSSI. Furthermore, multivariate regression analysis revealed that NSSI frequency in the last month and CRP levels were suicidal ideation predictors in adolescent MDD with NSSI, which may indicate that the increased frequency of NSSI behavior is a potential risk factor for suicide. Additionally, we explored the correlation between psychological and blood biochemical indicators to distinguish suicide attempters among participants having adolescent MDD with NSSI and identified a unique correlation network that could serve as a marker for suicide attempters. Our research data further suggested a complex correlation between the psychological and behavioral indicators of impulsivity and anger. Therefore, our study findings may provide clues to identify good clinical warning signs for SA in patients with adolescent MDD with NSSI.

## Introduction

Major depressive disorder (MDD) is the leading cause of disability worldwide and is considered the main contributor to the global disease burden in youth [1]. MDD is a common multifactorial, recurrent, and chronic psychological illness [2], making it one of the major risk factors for suicide attempt (SA) and non-suicidal self-injury (NSSI) [3]. NSSI, which has a high incidence in adult and adolescent patients and is associated with many psychiatric disorders, refers to behaviors resulting in deliberate destruction or damage to one’s body without a conscious suicidal intention [4–6].

Suicide is a serious consequence of mental illness and is the fourth leading cause of adolescent death. According to the WHO (2021), more than 700,000 people die by suicide every year globally (Suicide worldwide in 2019: Global Health Estimates). Additionally, more than 90% of suicides occur in the context of depression, such as MDD. Furthermore, depression severity is positively correlated with NSSI probability [7] and exerts a mediating effect on stressful life events and NSSI [8, 9]. Therefore, individuals having MDD with NSSI are considered a potential suicidal population. Moreover, NSSI is highly prevalent and frequently co-occurs with SAs in the clinical and non-clinical adolescent populations, indicating its predictive ability for future SAs [10]. Considering that suicidal behavior usually begins with self-injury, a common behavior among young patients with depression, investigating the suicidal risk factors in people having MDD with self-injury behavior is critical. Based on the assessment of various behavioral abnormalities, such as executive dysfunction in adolescents with depression and NSSI [11] and patients having bipolar disorder with suicidal ideation [12] as well as impaired decision-making in those with SA [13], several statistical and actuarial scales have been developed to aid clinicians to predict and manage suicide risk [14–16]. Studies investigating biomarkers and neural markers of suicide risk [17] have suggested neural connectivity as a response predictor for psychotherapy in individuals with NSSI [18] as well as biologically-informed approaches [19], including low serum cholesterol level, as potential suicide risk biomarkers in patients with MDD and suicidal behavior [2, 20, 21]. Blunted hypothalamic–pituitary–adrenal (HPA) axis activity [22, 23] and dysfunctional adaptive immune responses [24] are also associated with increased SAs. Additionally, elevated inflammation, as demonstrated by increased serum CRP levels, has been linked to patients with SA [25, 26], while dyslipidemia is associated with suicide risk [27].

Massive studies have examined the differences in the psychological, immunological, and metabolic characteristics between patients with NSSI and those with suicidal ideation, revealing a correlation between the immunological and psychological properties in patients with SA [11]. However, studies mainly focusing on the clinical differences between patients with suicidal and non-suicidal behavior have generally noted inconsistent results owing to the limited reliability of the univariate analyses employed for the heterogeneous clinical samples.

In this study, we aimed to investigate the differences and correlation networks of various psychological and biochemical indicators of adolescents having MDD and NSSI with SAs and those without SAs. The distinct correlation profile obtained in this study may offer clues for predicting suicide risk in patients having adolescent MDD with NSSI.

## Participants and methods

### Participants

In this study, G·Power software was used to estimate the sample size, and T-test of two independent samples was used as the test family. The effect size was set at 0.5, the error possibility at 0.05, the power at 0.8 and the distribution ratio at 1. The sample size of both groups was 64. We recruited 163 participants with major depression associated with NSSI from the Department of Depressive Disorders, Shenzhen Kangning Hospital between March 2020 and January 2021 in the study. Participants were young adults aged 12–35 years old. 33 patients were excluded, including 12 with comorbidities of anxiety disorder, three with endocrine system diseases, eight without completing blood test, seven unable to complete scale assessment, and three with lipid-lowering medications. The study finally recruited 130 participants, all of whom were diagnosed with major depressive disorder by two trained psychiatrists according to the DSM-5 diagnostic criteria and had a history of at least one year of self-injury, and 80 of those who had reported SA in the last year were classified as the group of suicide attempter (“NSSI + SA”), while the remaining 50 were classified as NSSI group. All participants were taking selective serotonin reuptake inhibitors as antidepressants. All participants had no major physical illness (including no acute or chronic infectious diseases or heart, liver, kidney, endocrine, or immune system diseases) or other psychological disorder. Additionally, none of the participants reported using any immunosuppressants or glucocorticoids, which might significantly affect their immune function and hormone secretion, or any lipid- or sugar-lowering medication. Participants were excluded if they met any of the conditions mentioned above. The data collected from the selected participants consisted of questionnaire data including scores on the Ottawa Self-Injury Inventory (OSI), Beck Depression Inventory-II (BDI-II), State-Trait Anxiety Inventory (STAI), Barratt Impulsiveness Scale (BIS-11), Difficulties in Emotion Regulation Scale (DERS), State-Trait Anger Expression Inventory (STAXI-2), Borderline Symptom List (BSL-23), Childhood Trauma Questionnaire (CTQ), Eysenck personality Questionnaire (EPQ), Defense Style Questionnaire (DSQ), and Coping Style Questionnaire (CSQ) as well as biochemical assessment data using peripheral blood samples. This study was approved by the Ethical Committee of Shenzhen Kangning Hospital (Approval No. 2020-K033-01). Written informed consent was obtained from all included participants after providing them with information on the study procedures. Detailed information of all participants is displayed in Tables 1–3.

**Table 1:** Characteristics of the study participants and procedure.

Basic information	NSSI + SA (*n* = 80, 61.54%)	NSSI (*n* = 50, 38.46%)	*P* value
Age (SD)	20.20 (4.75)	22.32 (4.51)	0.014
Female, *n* (%)	69 (86.25%)	47 (94.0%)	0.166
Active smoking status, *n* (%)	33 (41.25%)	17 (34.0%)	0.408
Alcohol intake, *n* (%)	47 (58.75%)	25 (50.0%)	0.231
BMI	21.75 (4.92)	20.67 (3.38)	0.141
Age onset of MDD	15.55 (4.28)	16.68 (4.37)	0.149

*NSSI* *+* *SA* adolescent major depressive disorder with non-suicidal self-injury and suicide attempts, *NSSI* adolescent major depressive disorder with non-suicidal self-injury.

**Table 2:** Comparison of the psychological indicators in participants having MDD and NSSI with SAs and those without SAs.

Psychological indicators		NSSI + SA (*n* = 80, 61.54%) Mean ± SD	NSSI (*n* = 50, 38.46%) Mean ± SD	*t*-test *P* value
DSQ	Immature defense	5.63 (1.13)	5.49 (1.15)	0.492
	Mature defense	4.85 (1.24)	5.04 (0.97)	0.365
	Intermediate defense	4.92 (0.82)	4.94 (0.70)	0.920
CSQ	Trouble-shooting	0.45 (0.26)	0.50 (0.23)	0.307
	Rationalization	0.50 (0.19)	0.51 (0.21)	0.815
	Self-accusation	0.80 (0.17)	0.77 (0.18)	0.387
	Help-seeking	0.33 (0.22)	0.43 (0.24)	0.022
	Fantasy	0.62 (0.18)	0.60 (0.20)	0.437
	Withdraw	0.68 (0.18)	0.67 (0.18)	0.747
EPQ	Psychoticism (P)	59.49 (11.82)	56.56 (10.55)	0.158
	Internal and external propensity scale (E)	41.16 (15.41)	44.94 (10.15)	0.097
	Neuroticism (N)	68.91 (6.88)	69.34 (5.88)	0.718
BDI-II	Depression	34 (11.06)	32.52 (10.51)	0.450
BIS-11	BIS-11 total scores	76.51 (13.05)	71.90 (11.29)	0.043
	Attentional impulsiveness (AI)	21.45 (4.75)	19.84 (3.92)	0.049
	Motor impulsiveness (MI)	25.06 (5.25)	23.88 (4.89)	0.206
	Non-planning impulsiveness (NI)	30 (5.77)	27.9 (5.14)	0.039
STAI	State anxiety	60.13 (11.17)	58.84 (11.93)	0.545
	Trait anxiety	61.95 (8.62)	60 (9.44)	0.232
STAXI-2 Trait anger	Trait anger (TA)	28.53 (6.81)	27.34 (6.49)	0.331
	Angry reaction (TA-R)	11.48 (3.14)	11.3 (2.87)	0.752
	Anger temperament (TA-T)	11.91 (3.48)	11.34 (3.43)	0.364
STAXI-2 Status anger	State anger (SA)	28.78 (14.67)	24.58 (10.73)	0.065
	Anger feeling (SA-F)	10.45 (5.15)	8.98 (3.80)	0.066
	Anger verbally (SA-V)	9.68 (5.30)	8.36 (4.01)	0.114
	Anger physically (SA-P)	8.65 (4.86)	7.24 (3.66)	0.065
STAXI-2 Anger expression	Anger expression-in (AX-I)	23.1 (4.16)	22.78 (5.02)	0.709
	Anger expression-out (AX-O)	17.46 (5.20)	16.74 (4.26)	0.414
	Anger control-in (AC-I)	19.05 (5.59)	19.36 (4.46)	0.743
	Anger control-out (AC-O)	19.88 (5.39)	20.76 (4.52)	0.339
BSL-23	Borderline symptom (BSL-23)	54.1 (23.24)	50.38 (21.42)	0.366
CTQ	CTQ total scores	57 (15.33)	55.94 (14.69)	0.700
	Physical abuse	9.14 (5.06)	8.16 (4.29)	0.262
	Emotional abuse	13.9 (5.18)	12.9 (5.12)	0.288
	Sexual abuse	6.19 (2.88)	7.02 (2.85)	0.113
	Physical neglect	10.90 (4.16)	10.70 (4.20)	0.793
	Emotional neglect	16.86 (4.64)	17.16 (4.28)	0.717
DERS	DERS total scores	122.29 (24.75)	118.66 (22.14)	0.403
	Awareness	20.45 (4.52)	19.74 (5.00)	0.409
	Clarity	14.65 (4.46)	13.04 (3.78)	0.037
	Non-acceptance	18.66 (6.56)	18 (5.75)	0.561
	Impulse	20.35 (6.28)	19.04 (5.91)	0.242
	Goals	19.73 (4.11)	19.3 (3.86)	0.562
	Strategies	28.45 (7.29)	29.54 (7.20)	0.410

*MDD* Major depressive disorder, *NSSI* *+* *SA* adolescent major depressive disorder with non-suicidal self-injury and suicide attempts, *NSSI* adolescent major depressive disorder with non-suicidal self-injury.

**Table 3:** Comparison of the blood biochemical indexes of participants having MDD and NSSI with SAs and those without SAs.

Biochemical indicators	NSSI + SA (*n* = 80, 61.54%) Mean ± SD	NSSI (*n* = 50, 38.46%) Mean ± SD	*t*-test *P* value
Glucose, mean	4.82 (0.42)	4.79 (0.56)	0.666
TG, mean	1.24 (0.60)	1.20 (0.81)	0.786
TC, mean	4.55 (0.68)	4.64 (0.89)	0.516
HDL, mean	1.56 (0.33)	1.61 (0.36)	0.418
LDL, mean	2.37 (0.53)	2.41 (0.65)	0.707
Cortisol, mean	242.58 (130.80)	293.26 (106.76)	0.024
ACTH, mean	15.91 (14.46)	14.93 (12.49)	0.694
CRP, mean	2.32 (0.60)	2.63 (0.61)	0.005

*TG* triglyceride, *TC* total cholesterol, *HDL* high-density lipoprotein, *LDL* low-density lipoprotein, *ACTH* adrenocorticotropic hormone, *CRP* C-reactive protein, *MDD* Major depressive disorder, *NSSI* *+* *SA* adolescent major depressive disorder with non-suicidal self-injury and suicide attempts, *NSSI* adolescent major depressive disorder with non-suicidal self-injury.

### Methods

#### Evaluation of biochemical and immunological indicators

Blood samples of the participants were collected between 7:00 and 8:00 a.m., after 12‑h overnight fasting. A total of 5 mL of venous blood was extracted and centrifuged at 2500 rpm for 10 min. The serum obtained as the supernatant was then used for further analysis. Enzyme-linked immunosorbent assay methods were used for determining the serum concentrations of lipids including triglyceride (TG), total cholesterol (TC), high density lipoprotein (HDL) and low density lipoprotein (LDL), cortisol, and blood glucose, whereas the CRP and Adrenocorticotropic Hormone (ACTH) levels were measured via immunoturbidimetry.

#### Psychological assessment

##### Ottawa Self-Injury Scale (OSI)

The OSI is used to assess NSSI behavior, consisting of a series of independent subscales to assess the intention and frequency of NSSI behavior, the motivation for initial and ongoing NSSI behavior, and addiction and other NSSI behavioral characteristics [28]. In this study, we utilized the first OSI item to evaluate the frequency of NSSI in the past year and the last month, while the second item was employed to check whether the participant had suicidal behavior.

##### Beck Depression Inventory-II (BDI-II)

BDI-II is a commonly used, well-validated self-report measure of depressive symptoms, with the depression levels determined based on the scores. The Chinese version of BDI-II has been widely administered to assess depression symptoms and severity in patients with mental illness and the general population [29].

##### State-Trait Anxiety Inventory (STAI)

The STAI comprises two subscales: one estimates state anxiety and the other detects trait anxiety. The total scores on each subscale range from 20 to 80, with higher scores indicating increased anxiety symptoms. The STAI has demonstrated good applicability in the Chinese population [30].

##### Barratt Impulsiveness Scale (BIS-11)

BIS-11 is applied to evaluate impulsivity in individual behavior. It includes three subscales (motor, attentional, and non-planning) that are summed to a total score of 30–120, with higher scores indicating higher impulsivity levels. BIS-11 has shown good psychometrics in Chinese population samples [31].

##### Difficulties in Emotion Regulation Scale (DERS)

The DERS is an effective instrument that gauges the difficulty in emotion regulation based on the following six factors: nonacceptance of emotional responses (Non-acceptance); difficulty engaging in goal-directed behavior (Goals); impulse control difficulties (Impulse); lack of emotional awareness (Awareness); limited access to emotion regulation strategies (Strategies); lack of emotional clarity (Clarity). A higher DERS score reflects greater difficulty in emotional regulation and lower emotional regulation ability. Studies investigating the DERS in the Chinese population have established its suitability [32].

##### State Trait Anger Expression Scale (STAXI-2)

STAXI-2 is currently the preferred tool for evaluating anger experience and expression and functions as a simple, objective scoring method for assessing the experience, expression, and control of anger. This inventory measures state anger, trait anger, and anger expression and control.

##### Borderline Symptom List (BSL-23)

BSL-23 [33] is a self-rating questionnaire to determine borderline symptoms, with a total score of 0–92. The severity of the borderline symptoms increases with increasing BSL-23 score. This instrument has exhibited good psychometrics in the Chinese population [34].

##### Childhood trauma questionnaire (CTQ)

The CTQ developed by Bernstein et al. is currently one of the most effective tools for measuring childhood abuse [35]. This assessment scale measures both abuse and neglect experiences. The abuse experiences cover emotional, physical, and sexual abuse, while the neglect experiences include emotional and physical neglect. The Chinese version of the CTQ has demonstrated favorable psychometric properties [36].

##### Eysenck personality Questionnaire (EPQ)

The Eysenck personality questionnaire (EPQ) was used to assess the participants’ personality factors. This study used the adult version of the Chinese version of the EPQ revised by Gong [37]. The scale contains 88 questions divided into four main factors: psychoticism, neuroticism, extraversion, and lie. The researchers used T-scores based on standardized data from the Chinese population - the higher the score of each factor, the higher the level of that personality trait of the individual.

##### Defense Style Questionnaire (DSQ)

The DSQ is a reliable and valid scale widely employed to evaluate the defense mechanisms by national and international studies owing to its suitability for people with different cultural backgrounds. The four subscales of this assessment tool include immature, mature, intermediate, and cover-up defense styles.

##### Coping Style Questionnaire (CSQ)

The CSQ was constructed by Chinese psychologists by incorporating the content of the questionnaires used by domestic and foreign experts to study coping and defensive behaviors, along with the theory of “coping”. The questionnaire comprises 62 items grouped into the six subscales of retreat, illusion, self-blame, help-seeking, rationalization, and problem-solving coping styles. Each item has two possible responses (“yes” or “no”), wherein a higher score in a particular subscale suggests that the respondent is more likely to respond in that corresponding coping style. The reliability and validity of the CSQ have been confirmed, and it is widely used in the Chinese population [38].

### Statistical analyses

The preliminary analysis examined the data for outliers and determined if the data met the assumptions of normality or linearity as appropriate. The independent samples *t*-tests were performed to compared all the indicators between two groups. Before comparing the biochemical and psychological indicators of the two groups using the t-test, we estimated the variance of indicators of each group and compared whether the variance of the two groups was similar. The Pearson correlations were performed to determine the inter-relationships between the various indicators via MATLAB (MathWorks Inc., MA, US). The Benjamini–Hochberg FDR (false discovery rate)-control procedure was performed to adjust the *P*-values in heatmaps and undirected graph plots. Only those interrelationships with *q* < 0.05 were shown. Multivariate regression analysis was conducted by including all variables that were significantly associated with SA in the univariate analysis (*P* < 0.1) using the backward LR method (*P* < 0.05). *t*-test and regression analyses were performed using SPSS version 26.0 (SPSS Inc., Chicago, IL, US). Statistical significance was set at two-tailed *P* < 0.05.

## Results

### Participant characteristics

The demographic characteristics of all 130 participants diagnosed with MDD and NSSI are summarized in Table 1. Among them, 80 (61.54%) participants reported SAs (NSSI + SA group), and 50 (38.46%) only exhibited self-injury with no SAs (NSSI group). The higher proportion of participants in the NSSI + SA group than in the NSSI group suggests that patients having MDD accompanied with NSSI may be at high risk for suicidal behaviors. Furthermore, there was a significant difference in age between NSSI + SA group and NSSI group, the participants in the NSSI + SA group (mean age: 20.2 years) were 2.12 years younger than those in the NSSI group (*P* = 0.014). However, no significant differences were observed regarding sex (*P* = 0.166), active smoking status (*P* = 0.408), alcohol intake (*P* = 0.231), BMI (*P* = 0.141) and age onset of MDD (*P* = 0.149) between the two groups. Finally, higher proportions of female participants versus male participants were observed in both the NSSI + SA and NSSI groups.

### Clinical differences between participants in the NSSI + SA and NSSI groups

Table 2 shows the differences between the NSSI + SA and NSSI groups in the psychological indicators based on the scores of multiple behavioral assessments. From the 11 clinical scales mentioned earlier, we derived 44 psychological indicators to comprehensively assess the psychological indicators of the participants with MDD and NSSI. The results showed that help-seeking (*P* = 0.022), BIS-11 total score (*P* = 0.043), attentional impulsiveness (*P* = 0.049), non-planning impulsiveness (*P* = 0.039), and lack of emotional clarity (*P* = 0.037) were significantly different between the NSSI + SA and NSSI groups. Except for lack of emotional clarity and help seeking, the other three psychological indicators were related to impulsiveness, consistent with previous research linking impulsivity with self-harm behaviors [39]. Therefore, our results indicated that participants in the NSSI + SA group were more impulsive than those in the NSSI group.

### Lower serum CRP and cortisol levels in the NSSI + SA group than in the NSSI group

The values of the biochemical indicators determined using the blood samples are shown in Table 3. The mean glucose levels were 4.82 ± 0.42 mmol/L and 4.79 ± 0.56 mmol/L (*P* = 0.666), while the mean TG concentrations were 1.24 ± 0.60 mmol/L and 1.20 ± 0.81 mmol/L (*P* = 0.786) in the NSSI + SA and NSSI groups, respectively, although these differences were statistically insignificant. Additionally, no significant differences in the TC, HDL, LDL, and ACTH levels were present between the two groups. However, the serum cortisol (*P* = 0.024) and CRP (*P* = 0.005) levels were significantly lower in the NSSI + SA group than in the NSSI group.

### Correlation networks of psychological and biochemical indicators in the NSSI + SA and NSSI groups

**Fig. 1:**
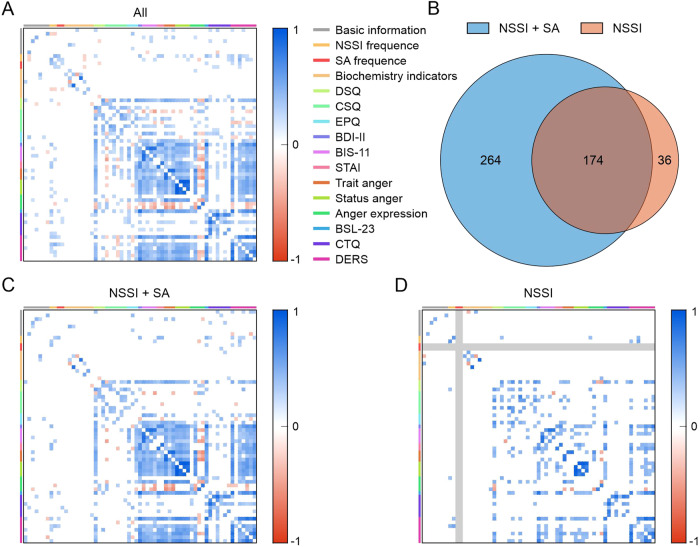
Heatmaps showing the Pearson correlation coefficients between all clinical indicators in all 130 participants with MDD and NSSI (FDR adjusted *P* < 0.05). The parameters used for the correlation analyses in the heatmaps are listed in Supplementary Table 2. **A** Pairwise comparisons of all behavioral indexes and biochemical indicators are shown in color bars on the top, with the Pearson correlation coefficient *R* color-coded according to the color bar on the right. All 63 indicators included basic characteristics and biochemical and psychological indicators (see Supplementary Table 2). **B** A total of 438 correlations were detected (FDR adjusted *P* < 0.05) in the NSSI + SA group, and only 210 correlations were revealed in the NSSI group, with 174 correlations shared by the two groups. Distinct correlation profiles of the clinical characteristics of the NSSI + SA (**C**) and NSSI groups (**D**). MDD Major depressive disorder, NSSI + SA adolescent major depressive disorder with non-suicidal self-injury and suicide attempts, NSSI adolescent major depressive disorder with non-suicidal self-injury.

The regression analysis results showed that self-injury frequency (a psychological indicator) in the past month (odds ratio [OR]: 1.588, 95% confidence interval [CI]: 1.006–2.508, *P* = 0.047) and serum CRP level (a biochemical indicator) were predictors of suicidal behavior in the participants having MDD with NSSI (OR: 0.392, 95% CI: 0.200–0.767, *P* = 0.006; Supplementary Table 1). Further, a correlation analysis was performed among the 63 indicators, including the basic characteristics and biochemical and psychological indicators, in the NSSI + SA and NSSI groups (Fig. 1A; Supplementary Table 2). As a result, 438 correlations were detected in the NSSI + SA group, whereas only 210 correlations were detected in the NSSI group (Fig. 1B). In addition, the Spearman correlation heatmaps indicated the presence of linear correlations between specific indicators and unique correlation heatmaps for the NSSI + SA and NSSI groups (Fig. 1C, D).

**Fig. 2:**
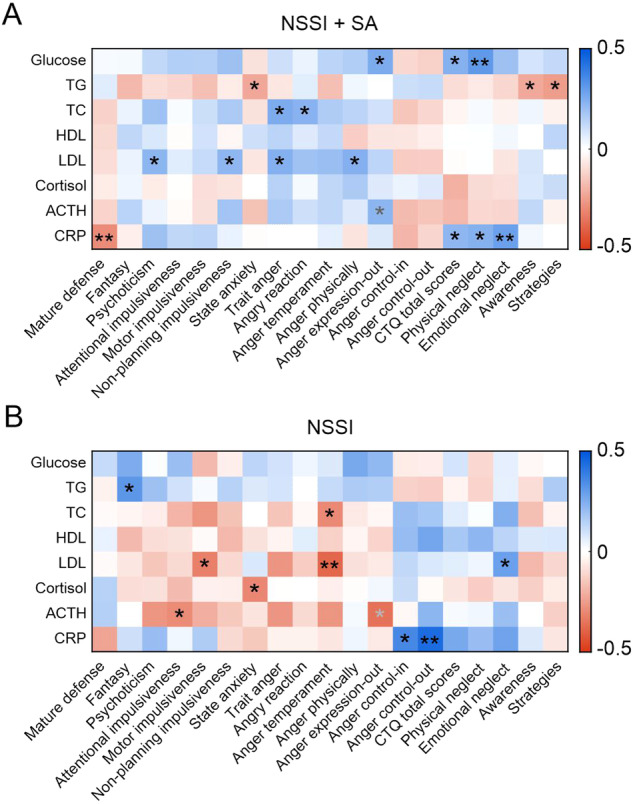
Correlation heatmaps of the psychological and biochemical indicators. Heatmaps presenting the Pearson correlation coefficients between the psychological and biochemical indicators in the NSSI + SA (**A**) and NSSI groups (**B**) among the participants with adolescent MDD and NSSI. Psychological indicators that significantly correlated with at least one biochemical indicator in either one or both groups are shown. The *R* values are color-coded based on the color bar on the right. *P < 0.05 and **P < 0.01. The asterisk in black indicates the specific correlations within each group, while the asterisk in gray represents the correlations shared between the two groups (*P* < 0.05). MDD Major depressive disorder, NSSI + SA adolescent major depressive disorder with non-suicidal self-injury and suicide attempts, NSSI adolescent major depressive disorder with non-suicidal self-injury.

**Fig. 3:**
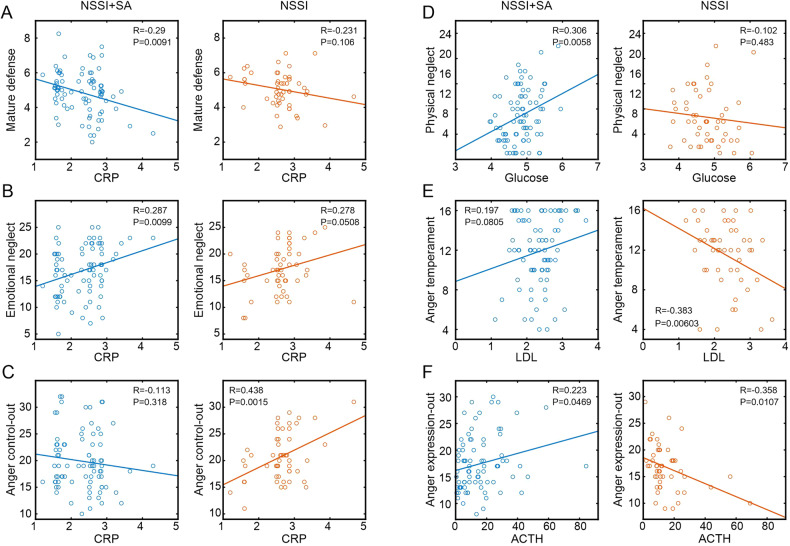
Scatter plots depicting the representative correlations between the psychological and biochemical indicators in the NSSI + SA and NSSI groups among participants with adolescent MDD and NSSI. Distinct linear correlations are observed between CRP level and mature defense (**A**), CRP level and emotional neglect (**B**), CRP level and anger control-out (**C**), glucose concentration and physical neglect (**D**), LDL values and anger temperament (**E**), and ACTH level and anger expression-out (**F**). R and *P* stand for the Pearson correlation coefficient and the corresponding *P* value, respectively. MDD Major depressive disorder, NSSI + SA adolescent major depressive disorder with non-suicidal self-injury and suicide attempts, NSSI adolescent major depressive disorder with non-suicidal self-injury.

Thus, we further explored the linear correlations between the biochemical and psychological indicators and the specific correlation heatmaps for the two groups (Fig. 2A, B). CRP level demonstrated a significant negative correlation with mature defense (*R* = − 0.29, *P* = 0.0091) and a significant positive association with emotional neglect (*R* = 0.287, *P* = 0.0099) in the NSSI + SA group, whereas no such relationships were found in the NSSI group (*P* = 0.106, Fig. 3A and P = 0.0508, Fig. 3B, respectively). However, CRP level did show a positive correlation with anger control-out in the NSSI group (*R* = 0.438, *P* = 0.0015), which was not observed in the NSSI + SA group (*P* = 0.318, Fig. 3C). In addition, glucose concentration exhibited a positive association with physical neglect (*R* = 0.306, *P* = 0.0058) in the NSSI + SA group, but no such link was observed in the NSSI group (*P* = 0.483, Fig. 3D). Additionally, LDL level and anger temperament were negatively correlated in the NSSI group (R = −0.383, *P* = 0.00603, Fig. 3E) but not in the NSSI + SA group. Finally, ACTH concentration demonstrated a positive association with anger expression-out in the NSSI + SA group (*R* = 0.223, *P* = 0.0469) and a negative correlation with anger expression-out in the NSSI group (*R* = − 0.358, *P* = 0.0107, Fig. 3F).

**Fig. 4:**
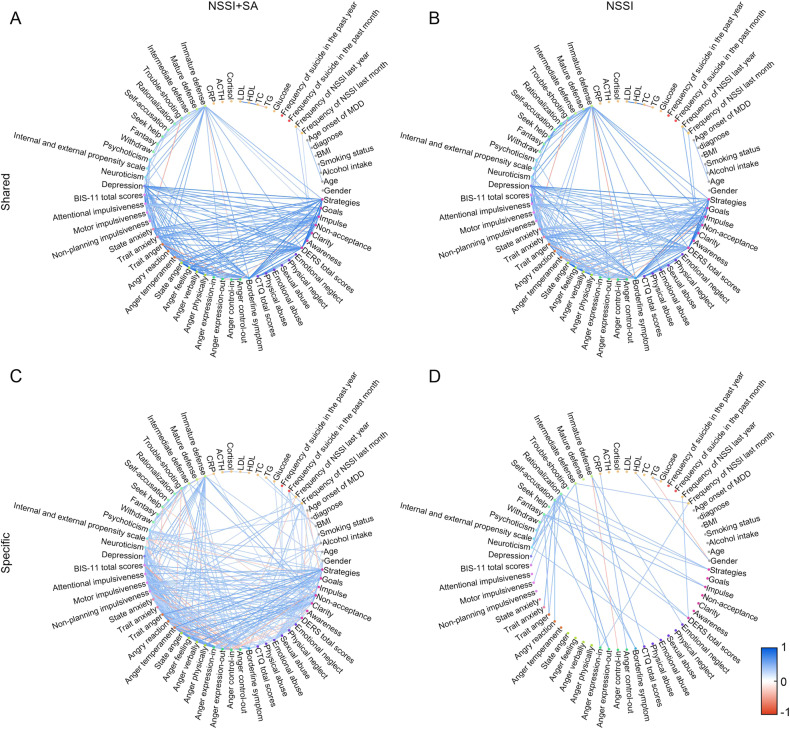
Association networks of the NSSI + SA and NSSI groups. Undirected graphs showing the common (**A**, **B**) and unique (**C**, **D**) characteristics of the association networks of the NSSI + SA and NSSI groups among participants with adolescent MDD and NSSI (FDR adjusted *P* < 0.05). The Pearson correlation coefficients are color-coded according to the color bar on the bottom right. MDD Major depressive disorder, NSSI + SA adolescent major depressive disorder with non-suicidal self-injury and suicide attempts, NSSI adolescent major depressive disorder with non-suicidal self-injury.

**Fig. 5:**
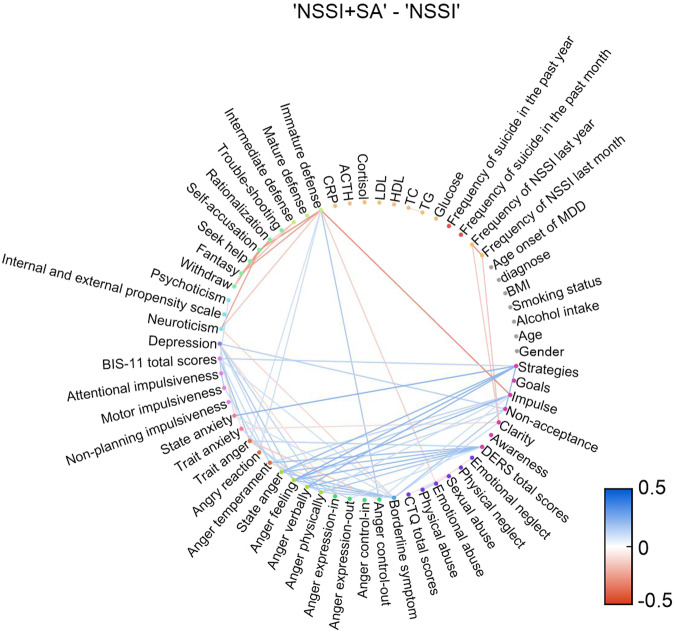
Undirected graph of the differential association networks illustrating the differences between the common characteristics of the NSSI + SA and NSSI groups in participants with adolescent MDD and NSSI (FDR adjusted *P* < 0.05). Differences are color-coded according to the color bar at the bottom right. MDD Major depressive disorder, NSSI + SA adolescent major depressive disorder with non-suicidal self-injury and suicide attempts, NSSI adolescent major depressive disorder with non-suicidal self-injury.

To further examine the distinct association network features in the NSSI + SA and NSSI groups, we analyzed the common (Fig. 4A, B) and unique (Fig. 4C, D) characteristics of these association networks. Our findings showed that the psychological indicators related to the expression of anger, impulsivity, and immature defense showed more complex correlations in the NSSI + SA group than in the NSSI group. Further analysis of the differences between the association networks of the two groups highlighted that participants with SAs had strong correlations in the psychological indicators related to impulsivity and the expression of emotion or anger, as shown in Fig. 5. Overall, these correlation networks indicated the significance of impulsivity- and anger-related behavioral correlates in SA among patients having adolescent MDD with NSSI.

## Discussion

In this current study, we have demonstrated that the age of suicide attempters is lower than that of non-attempters in adolescent patients having MDD with NSSI; suicide attempters have higher score on impulsivity, while levels of cortisol and CRP are inversely correlated with suicide attempts in patients with adolescent MDD with NSSI. Our multivariate regression analysis indicate that the increased frequency of NSSI behavior is a potential risk factor for suicide. Most importantly, the correlation network of the psychological and biochemical indicators in suicide attempters indicated that more complex associations exist in the behavioral correlates of impulsivity and anger in patients having adolescent MDD and NSSI with SAs.

In recent years, NSSI and suicide have attracted attention as major public health concerns in young adults due to their growing trend in this age group with MDD [40, 41]. Along with the extensive studies on the psychosocial correlates of NSSI and SAs [42], the longitudinal risk factors for NSSI and SA have also been recently researched [43–46]. However, NSSI and SAs are distinct behaviors in terms of clinical presentation and history, despite having a high co-occurrence rate in adolescents and young adults. Researchers have comprehensively investigated the association between NSSI and SAs in young adults, with some studies using behavioral characterization to distinguish self-injury and suicide risk in young patients with MDD [47]. However, scarce investigations have explored the biochemical markers that might screen suicide attempters among patients with NSSI. Moreover, the conclusions drawn from these examinations could vary owing to the limited sample sizes and individual differences among patients with complex psychosis. In this study, we thoroughly analyzed the psycho-behavioral and blood biochemical indicators in participants having MDD accompanied with NSSI and further investigated the differences between those with and without SAs.

Our findings suggested that the age of suicide attempters was lower than that of non-attempters among participants with MDD and NSSI, which was in line with previous study results [14–16]. These findings indicate that more attention must be paid to the young population having MDD with NSSI when assessing potential suicidal behavior. Furthermore, we found that self-injury frequencies in the NSSI + SA group in the past month and year were significantly higher than those in the NSSI group, suggesting that increased frequency of NSSI behavior may be a potential risk factor for suicidal behavior. This observation was further confirmed by our regression analysis, which demonstrated that self-injury frequency in the past month was a potential predictor for suicidal behavior. Our results offer evidence for clinicians that interventions for reducing NSSIs in participants with MDD may prevent the occurrence of suicidal behaviors.

Our study also showed that five out of the 44 psychological indicators significantly differed between suicide attempters and non-attempters among participants with MDD and NSSI. These indicators included help-seeking, BIS-11 total score, attentional impulsiveness, non-planning impulsiveness, and lack of emotional clarity, among which three were related to impulsivity. Considering that impulsivity has a close, complex relationship with NSSI, adolescents with trait impulsivity, such as negative impulsiveness, are more likely to exhibit NSSI [48]. Other studies have also reported that increased impulsivity might be a potential risk factor for NSSI, implying that impulsivity might be an independent predictor for NSSI [49–51]. In our study, BIS-11 total score, attentional impulsiveness, and non-planning impulsiveness were significantly higher in suicide attempters than in non-attempters. Moreover, our results indicated that impulsivity could function as an independent risk factor for suicidal behaviors, consistent with the findings from previous reports [17–19]. However, previous work generally focused on suicide attempters without considering the complex conditions of patients with mental disorders. Therefore, those results may require further examination. Nevertheless, Zakowicz et al. found no significant difference in the BIS-11 scores between suicide attempters and non-suicidal patients with bipolar disorder [52].

Furthermore, we investigated the potential biological basis of the differences between participants with and without SAs among those with MDD and NSSI. We revealed that both cortisol and CRP levels were significantly lower in suicide attempters than in non-attempters. Additional logistic regression analysis demonstrated that CRP level was a predictor for suicidal behavior in participants having MDD with NSSI. Studies have shown that the HPA axis is the major stress response system in rodents, and its role in psychopathology has been extensively examined [29]. Moreover, cortisol level is utilized to assess the function of the HPA axis because the HPA axis regulates cortisol secretion by the adrenal cortex. Although stress exposure is central to the theories of suicide and potential social-environmental triggers of suicide have been elucidated [53], biological characteristics, such as serum cortisol level, might also be useful predictors of suicide risk [54]. In our study, serum cortisol level was significantly lower in suicide attempters than in non-attempters, supporting the previous notion of the role of reduced HPA axis activity and cortisol levels in suicide attempters [2, 31–35]. In the case of serum CRP levels, one study reported significantly increased CRP levels in patients with MDD and suicidal behavior compared with patients with MDD and non-suicidal behavior and healthy controls [55]. However, another study by Orsolini et al. found that about one-third of patients with depression exhibited a low-grade inflammatory state in their research [56], consistent with our current finding of lower CRP levels in suicide attempters than in non-attempters. These discrepant results may be because of the different comparisons across the various study groups. For example, our study investigated the differences between suicide attempters and non-attempters within the patient population having MDD and NSSI. All these data suggest that abnormal changes in blood biochemistry in response to stress are associated with suicidal ideation and SAs in patients having adolescent MDD and NSSI. However, applying a single behavioral or biochemical characteristic as a predictor for suicidal behavior in complex cases of MDD with NSSI may not be adequate; thus, multi-parameter monitoring is necessary for suicide prediction in patients with mental disorders.

To address this need for multiple predictors of suicide risk, we analyzed the correlation profiles of the psychological and biochemical indicators in the NSSI + SA and NSSI groups. We found a correlation profile that was unique to suicide attempters compared with non-attempters. Our results demonstrated significant differences in the CRP levels as well as correlations between the CRP levels and psychological indicators among the participants with and without SAs. Particularly, serum CRP level was negatively correlated with mature defense and positively correlated with emotional neglect in suicide attempters, while it was positively correlated with anger control-out in non-attempters. These findings imply that the serum CRP level and psychological indicator scores may have differential interactions in patients with MDD and NSSI based on their experience of SA. Therefore, our results suggest that serum CRP level may be a key monitoring tool for suicidal ideation in MDD with NSSI.

Furthermore, our study revealed that glucose concentration was positively correlated with physical neglect in suicide attempters, while LDL level was negatively correlated with anger temperament in non-attempters. Moreover, patients with MDD have been reported to present with metabolic dysfunction, including altered blood levels of glucose, insulin, and glucagon [57, 58]. One study found that participants having MDD with SAs had higher glucose levels than those without SAs [59], whereas another research indicated that serum LDL level was associated with depression severity [60]. Additionally, suicide attempters >40 years were demonstrated to have significantly higher LDL levels than non-attempters [61]. Therefore, serum LDL level may be a potential predictor of SAs in patients having MDD with NSSI, indicating that this parameter needs to be closely monitored, routinely screened, and regularly followed up in this patient population [62].

In this study, we aimed to investigate the predictors of SAs in participants having MDD with NSSI by analyzing the correlation profile of the blood biochemical and psychological indicators based on our notion that surveillance of any one factor may not be sufficient to predict suicidal behavior in the clinical setting. Apart from the impulsiveness scores, serum CRP and cortisol levels were shown to be potential risk factors for SAs in participants with MDD and NSSI. Further exploration of the differences between suicide attempters and non-attempters among participants with adolescent MDD and NSSI indicated that suicide attempters had a unique association network of psycho-behavioral and biochemical indicators that exhibited a more complex correlation. Our study findings also suggested that the psychological indicators related to anger and impulsivity in patients having adolescent MDD with NSSI require close clinical monitoring. Finally, the correlation profiles of the psychological and biochemical indicators examined in this study may provide insight into distinguishing suicide attempters among patients having adolescent MDD with NSSI in clinical settings. The limitation of our current study is that we here analyzed only 130 patients; continuous work is ongoing regarding the multivariate predictive models for predictors of SAs in patients having MDD with NSSI based on larger sample sizes. Given the intricate nature of neuropsychiatric disorders such as MDD and the trend towards using machine learning methods to identify disease subtypes and make predictions [63, 64], in our future research, machine learning techniques will be employed to integrate psycho-behavioral parameters with pathological indicators, including gut microbiota and blood multi-omics data, which will be based on multicentre studies with large clinical samples. This will undoubtedly enable the early identification and prevention of suicidal behaviors in adolescent MDD and NSSI.

In conclusion, our study suggests a complex association network between specific psychological and biochemical indicators, thereby extending the previous research findings that highlighted the potential role of biochemical indicators in predicting suicidal behavior. In particular, our results emphasize that the behavioral correlates for emotional expression may be a critical indicator for predicting suicidal behavior in patients having adolescent MDD with NSSI.

### Supplementary information

Supplemental Tables

## Data Availability

The authors confirm that the data supporting the findings of this study are available within the article and its supplementary materials.
