# Supplementary material for: Distinct correlation network of clinical characteristics in suicide attempters having adolescent major depressive disorder with non-suicidal self-injury
Source: Transl Psychiatry. 2024 Mar 5;14:134. doi: 10.1038/s41398-024-02843-w (PMC10914800; doi:10.1038/s41398-024-02843-w)
Supplement: Supplementary file 1 — Supplemental Tables [file 41398_2024_2843_MOESM1_ESM.docx]

**Supplemental Tables for**

**Distinct Correlation Network of Clinical Characteristics in Suicide Attempters Having Adolescent Major Depressive Disorder with Non-suicidal Self-Injury**

**Bo Peng^1, 2, #^, Ruoxi Wang^3, #^, Wenlong Zuo^4^****^, #^, Haitao Liu^5^, Chunshan Deng^3^,** **Xiaoyuan Jing^3^, Hongtao Hu^1, 2^, Weitan Zhao****^1, 2^, Peiwu Qin^6, 7^, Lei Dai^4, 8^, Zuxin Chen^3, 8, *^, Yingli Zhang^1, 2, *^, Xin-an Liu^3, 8^****^, *^**

**Supplemental Table. 1**

|  | Univariate analysis | | Multivariate analysis | |
| --- | --- | --- | --- | --- |
|  | OR (95% CL) | *P* | OR (95% CL) | *P* |
| Age | 0.909 (0.842,0.982) | 0.016 |  |  |
| Frequency of NSSI  last month | 1.701 (1.111,2.605) | 0.015 | 1.588 (1.006,2.508) | 0.047 |
| Frequency of NSSI  last year | 1.608 (1.086,2.382) | 0.018 |  |  |
| BIS-11 total scores | 1.030 (1.001,1.061) | 0.045 |  |  |
| Attentional impulsiveness (AI) | 1.084 (1.000,1.177) | 0.051 |  |  |
| Non-planning impulsiveness (NI) | 1.070 (1.003,1.143) | 0.042 |  |  |
| State anger (SA) | 1.025 (0.996,1.055) | 0.088 |  |  |
| Anger feeling (SA-F) | 1.071 (0.990,1.160) | 0.088 |  |  |
| Anger physically (SA-P) | 1.080 (0.989,1.179) | 0.088 |  |  |
| Clarity | 1.096 (1.004,1.197) | 0.040 |  |  |
| Cortisol | 0.997 (0.994,1.000) | 0.027 |  |  |
| CRP | 0.420 (0.223,0.768) | 0.007 | 0.392 (0.200,0.767) | 0.006 |

**Supplemental Table 1.** Logistic regression analysis of the clinical and biochemical variables for suicide attempts (SAs). Univariate analysis was performed to investigate the correlation between individual variables and SA. Multivariate regression analysis was conducted by including all variables that were significantly associated with SA in the univariate analysis (*P* < 0.1) using the Backward LR method (*P* < 0.05). MDD: Major depressive disorder. NSSI: non-suicidal self-injury. SA: suicide attempts.

**Supplemental Table. 2**

| 1 | Gender | 2 | Age |
| --- | --- | --- | --- |
| 3 | Alcohol intake | 4 | Smoking status |
| 5 | BMI | 6 | diagnose |
| 7 | Age onset | 8 | Frequency of NSSI last month |
| 9 | Frequency of NSSI last year | 10 | Frequency of suicide in the past month |
| 11 | Frequency of suicide in the past year | 12 | Glucose |
| 13 | TG | 14 | TC |
| 15 | HDL | 16 | LDL |
| 17 | Cortisol | 18 | ACTH |
| 19 | CRP | 20 | Immature defense |
| 21 | Mature defense | 22 | Intermediate defense |
| 23 | Trouble-shooting | 24 | Rationalization |
| 25 | Self-accusation | 26 | Help-seeking |
| 27 | Fantasy | 28 | Withdraw |
| 29 | Psychoticism | 30 | Internal and external propensity scale |
| 31 | Neuroticism | 32 | Depression |
| 33 | BIS-11 total scores | 34 | Attentional impulsiveness |
| 35 | Motor impulsiveness | 36 | Non-planning impulsiveness |
| 37 | State anxiety | 38 | Trait anxiety |
| 39 | Trait anger | 40 | Angry reaction |
| 41 | Anger temperament | 42 | State anger |
| 43 | Anger feeling | 44 | Anger verbally |
| 45 | Anger physically | 46 | Anger expression-in |
| 47 | Anger expression-out | 48 | Anger control-in |
| 49 | Anger control-out | 50 | Borderline symptom |
| 51 | CTQ total scores | 52 | Physical abuse |
| 53 | Emotional abuse | 54 | sexual abuse |
| 55 | Physical neglect | 56 | Emotional neglect |
| 57 | DERS total scores | 58 | Awareness |
| 59 | Clarity | 60 | Non-acceptance |
| 61 | Impulse | 62 | Goals |
| 63 | Strategies |  |  |

**Supplemental Table 2.** The parameters used in the correlation analyses for the heatmaps displayed in Figure 1.
